# OA17 Combination therapy as treatment for resistant Evans syndrome in a systemic lupus erythematosus patient

**DOI:** 10.1093/rap/rkae117.017

**Published:** 2024-11-05

**Authors:** Alana Jacobs, Matthew Pickering, Nichola Cooper

**Affiliations:** Hammersmith hospital, Imperial college healthcare NHS trust, London, United Kingdom; Hammersmith hospital, Imperial college healthcare NHS trust, London, United Kingdom; Hammersmith hospital, Imperial college healthcare NHS trust, London, United Kingdom

## Abstract

**Introduction:**

This is a case report of a complex SLE patient with pronounced haematological manifestations. Her haematological diagnoses of immune thrombocytopaenia (ITP) and autoimmune haemolytic anaemia (AIHA) preceded that of her lupus diagnosis. Her SLE was diagnosed on the basis of positive serology with haematological involvement, arthralgia and Raynaud’s. She was subsequently diagnosed with antiphospholipid syndrome (triple positive antiphospholipid antibodies) with arterial thromboses and previous miscarriages. Her disease has been resistant to routine management and has required treatment escalation as well as combination therapy.

**Case description:**

This case involves a Caucasian lady with a pre-existing diagnosis of ITP from over twenty years ago. More recently she had been diagnosed with AIHA conferring a diagnosis of Evans syndrome. Her AIHA was initially managed with high dose glucocorticoids plus rituximab via haematology. She was referred to rheumatology on the discovery of a strongly positive antinuclear antibody which raised the possibility of an underlying connective tissue disorder. On rheumatology review, she described new inflammatory joint symptoms as well as Raynaud’s phenomenon. Further blood work demonstrated positive dsDNA, low C3/4 plus RNP and SM antibodies. She reported three previous miscarriages but no thromboembolic events. She was ascertained to be triple positive on antiphospholipid antibody screen. Her initial lupus management was that of hydroxychloroquine, mycophenolate mofetil (MMF) plus low dose prednisolone. Despite this treatment she developed worsening thrombocytopaenia which failed to respond to increasing doses of steroids and IVIG. With multi-disciplinary team (MDT) consensus she was treated with an extended regimen of cyclophosphamide with good improvement to SLE serological markers. She required iloprost for her Raynaud’s during this period. For ongoing ITP, eltrombopag was initiated by haematology. She developed a DVT with a small non-occlusive PE which was treated with heparin and subsequently apixaban. Following a relapse of her AIHA, she was commenced on belimumab (intravenously and then subcutaneously). She continued to develop episodes of worsening anaemia and thrombocytopenia which were managed with steroid dose escalation, IVIG and intermittent cessation of apixaban. Unfortunately, she later suffered from bilateral pulmonary emboli requiring initiation of warfarin. For wider B-cell depletion, it was decided to start treatment with ofatumumab. Nevertheless, her AIHA once again flared and given her recent B cell depletion and hypocomplementaemia, MDT consensus was that for re-initiation of belimumab infusions. With this combination of treatment, her blood counts have stabilised.

**Discussion:**

This case highlights the complexity of managing haematological manifestations of SLE. The patient’s lupus driven ITP and AIHA have been refractory to conventional treatments requiring escalation of medication and combination therapy. Her management has required the targeting of several of the pathogenic pathways of SLE to control her disease. Treatment has involved glucocorticoids, hydroxychloroquine, MMF, cyclophosphamide, B cell depletion [rituximab, then later ofatumumab], belimumab, IVIG and eltrombopag. Adding to the intricacy of this case was the fact that the patient was triple positive for antiphospholipid antibodies with subsequent development of thrombotic phenomena. Balancing clot risk with anaemia and thrombocytopaenia has been most challenging. Successful management of the patient has been made possible through the close working with haematology and ongoing constant dialogue. It is notable that haematological symptoms can predate a diagnosis of SLE. This suggests the need to screen patients for secondary causes of AIHA, thrombocytopaenia and leukopaenia. AIHA is found to occur in around 10% of SLE patients. Thrombocytopaenia occurs more commonly and can be seen in up to 50% of cases (although is generally mild); around 10% of patients have severe thrombocytopaenia. Evans syndrome (diagnosed when two or more cytopaenias are present) is a much rarer complication that is seen in the lupus cohort. Notwithstanding the use of advanced and combination therapy, the patient has still required a large burden of glucocorticoids which renders the questions as to whether future therapies can offer a lower or even zero exposure to steroids to absolve the complications that these proffer.

**Key learning points:**

• Haematological abnormalities are often found in SLE patients and can predate diagnosis. The haematological manifestations of SLE are protean from mild thrombocytopenia to AIHA and myelofibrosis. Haematological disease may be a consequence of the disease but also can be attributed at times to the immunosuppressive treatments used in SLE. Thrombocytopenia has been noted to be associated with more severe SLE and poorer prognosis. Evans syndrome is diagnosed when two or more cytopenias are present which generally include AIHA and ITP. First line treatment for SLE haematological disease remains that of glucocorticoids but this will rarely result in a sustained response with the need for additional immunosuppressive treatments (often as combination therapy). Treatments include that of csDMARDS (such as MMF or azathioprine), IVIG, B-cell depletion, belimumab and cyclophosphamide. Splenectomy as treatment for ITP and AIHA is now rarely advised due to the infection risk it confers and variable remission rates. Combination therapy of belimumab with rituximab was examined by Mehevas et al. for ITP in a phase IIb trial on the basis that B-cell activating factor could be implicated in the failure of B-cell depletion treatment for encouraging the development of long-lasting splenic plasma cells. This trial demonstrated a reduction in circulating T-follicular helper cells with combination therapy with improved treatment response and acceptable safety.

